# Placental Protein 13 (PP13) – A Placental Immunoregulatory Galectin Protecting Pregnancy

**DOI:** 10.3389/fimmu.2014.00348

**Published:** 2014-08-20

**Authors:** Nándor Gábor Than, Andrea Balogh, Roberto Romero, Éva Kárpáti, Offer Erez, András Szilágyi, Ilona Kovalszky, Marei Sammar, Sveinbjorn Gizurarson, János Matkó, Péter Závodszky, Zoltán Papp, Hamutal Meiri

**Affiliations:** ^1^Perinatology Research Branch, Eunice Kennedy Shriver National Institute of Child Health and Human Development, National Institutes of Health, U.S. Department of Health and Human Services, Bethesda, MD, and Detroit, MI, USA; ^2^Department of Obstetrics and Gynecology, Wayne State University School of Medicine, Detroit, MI, USA; ^3^Maternity Private Department, Kútvölgyi Clinical Block, Semmelweis University, Budapest, Hungary; ^4^Institute of Enzymology, Research Centre for Natural Sciences, Hungarian Academy of Sciences, Budapest, Hungary; ^5^Department of Immunology, Eötvös Loránd University, Budapest, Hungary; ^6^Department of Obstetrics and Gynecology, Soroka University Medical Center, Ben-Gurion University of the Negev, Beer-Sheva, Israel; ^7^First Department of Pathology and Experimental Cancer Research, Semmelweis University, Budapest, Hungary; ^8^Prof. Ephraim Katzir Department of Biotechnology Engineering, ORT Braude College, Karmiel, Israel; ^9^Faculty of Pharmaceutical Sciences, School of Health Science, University of Iceland, Reykjavik, Iceland; ^10^TeleMarpe Ltd., Tel Aviv, Israel; ^11^Hylabs Ltd., Rehovot, Israel

**Keywords:** actin cytoskeleton, biomarker, danger signal, evolution, extracellular vesicles, glycans, lectins, maternal-fetal interface

## Abstract

Galectins are glycan-binding proteins that regulate innate and adaptive immune responses, and some confer maternal-fetal immune tolerance in eutherian mammals. A chromosome 19 cluster of galectins has emerged in anthropoid primates, species with deep placentation and long gestation. Three of the five human cluster galectins are solely expressed in the placenta, where they may confer additional immunoregulatory functions to enable deep placentation. One of these is galectin-13, also known as Placental Protein 13 (PP13). It has a “jelly-roll” fold, carbohydrate-recognition domain and sugar-binding preference resembling other mammalian galectins. PP13 is predominantly expressed by the syncytiotrophoblast and released from the placenta into the maternal circulation. Its ability to induce apoptosis of activated T cells *in vitro*, and to divert and kill T cells as well as macrophages in the maternal decidua *in situ*, suggests important immune functions. Indeed, mutations in the promoter and an exon of *LGALS13* presumably leading to altered or non-functional protein expression are associated with a higher frequency of preeclampsia and other obstetrical syndromes, which involve immune dysregulation. Moreover, decreased placental expression of PP13 and its low concentrations in first trimester maternal sera are associated with elevated risk of preeclampsia. Indeed, PP13 turned to be a good early biomarker to assess maternal risk for the subsequent development of pregnancy complications caused by impaired placentation. Due to the ischemic placental stress in preterm preeclampsia, there is increased trophoblastic shedding of PP13 immunopositive microvesicles starting in the second trimester, which leads to high maternal blood PP13 concentrations. Our meta-analysis suggests that this phenomenon may enable the potential use of PP13 in directing patient management near to or at the time of delivery. Recent findings on the beneficial effects of PP13 on decreasing blood pressure due to vasodilatation in pregnant animals suggest its therapeutic potential in preeclampsia.

## Preface

Many authors of this review have collaborated with Dr. Hans Bohn, the discoverer of Placental Protein 13 (PP13), who passed away on January 25, 2014. We dedicate this manuscript to his memory. His scientific legacy and enormous contribution to placental protein research have strongly influenced placentology and inspired our studies on PP13 (1).

**Figure 1:**
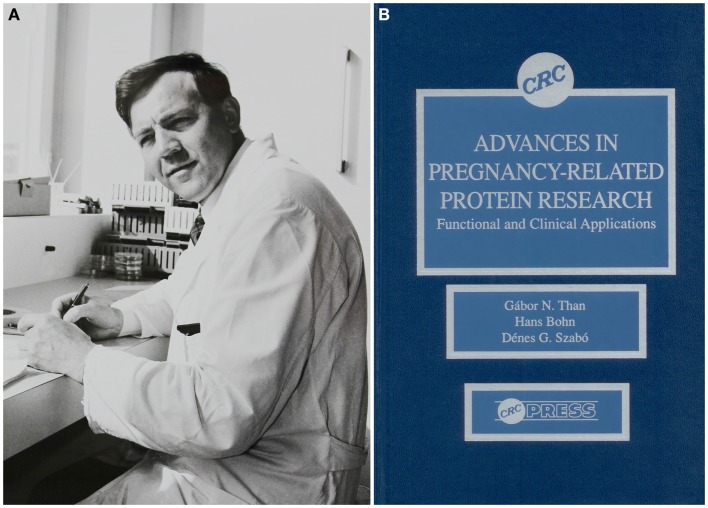
**Dr. Hans Bohn and his scientific legacy in pregnancy-related protein research**. **(A)** Dr. Hans Bohn is depicted in his Protein Laboratory at Behringwerke (Marburg/Lahn, Germany), where he discovered and isolated more than 50 placental, endometrial and pregnancy serum proteins including Placental Protein 13 (PP13), and developed specific antisera and immunosassays for them between 1965 and 1989. The photo was obtained as a courtesy from Gabriele Bohn. **(B)** A book co-written by Hans Bohn, Gábor N. Than and Dénes G. Szabó published in the USA summarized the existing knowledge in the research field in 1993.

Hans Bohn was born in Munich on October 18, 1928. He graduated in 1954 and completed his doctoral thesis in 1956 in chemistry at the University of Würzburg. A research fellowship starting in 1963 in the Protein Research Laboratory at the University of Pittsburgh was critical in directing his interest in protein research. After two years, Dr. Bohn returned to Germany to work on proteins in Behringwerke in Marburg/Lahn (Figure 1A). He was the first to isolate factor XIII from human placenta for the treatment of patients with factor XIII deficiency and wounds after injury or surgery, and a side-fraction of this experiment yielded human placental lactogen. This experience strongly influenced him to focus his research on the systematic isolation and characterization of placental, endometrial and pregnancy serum proteins. These studies have greatly supported our knowledge on pregnancy-related proteins and their application in the diagnosis of pregnancy complications (1).

Dr. Hans Bohn processed large amounts of human placental, amniotic fluid and serum specimens and utilized combinations of classical fractionation techniques to isolate more than 50 proteins, which he named sequentially. He characterized these proteins for their physico-chemical characteristics, and then developed specific rabbit antisera against them for further protein purification and for the development of immunoassays to determine these proteins’ diagnostic significance. In collaboration with scientists around the world, Dr. Bohn also determined the amino acid sequence as well as biological functions of many of these. Among the proteins he isolated were Placental Protein (PP) 4 (annexin-V), PP5 (tissue factor pathway inhibitor-2, TFPI-2), PP10 (plasminogen activator inhibitor-2, PAI-2), PP12 (insulin-like growth factor binding protein-1, IGFBP-1) and PP13 (galectin-13), which were subsequently identified to be important regulators of the fundamental processes in pregnancy (1).

Dr. Bohn’s collaboration with Professor Gábor N. Than (University of Pécs, Pécs, Hungary) had a fundamental impact on the cloning, sequencing, structural, and molecular biological characterization of a large number of PPs including PP13, and their pioneering collaborative research significantly improved our understanding on the biological role and diagnostic significance of these proteins in pregnancy complications and malignancies. Beyond these scientific discoveries, their friendship and close collaboration strongly inspired a new generation of scientists. The existing knowledge and advancements in the field were summarized in their book entitled *Advances in Pregnancy-Related Protein Research*, co-written with Dr. Dénes G. Szabó in 1993 (2) (Figure 1B).

For Dr. Bohn’s founding research and discovery of PP14 (glycodelin), he shared the prestigious Abbott Award in 1997. His remarkable contributions to placental and pregnancy-related protein research were published in 198 research articles. Dr. Bohn continued to contribute to placental protein research after his retirement, and closely followed the studies implementing novel molecular and cellular biological techniques on the proteins he isolated, leading to further discoveries and improvements in clinical diagnostics and patient care (1).

Dr. Hans Bohn was an exceptional scientist, an enthusiastic catalyzer of collaborations and friendships who inspired many peers and followers. He was a wonderful, kind and charismatic person, a silent giant, who will be greatly missed.

## The Discovery and Molecular Characterization of PP13

### Isolation, purification and physico-chemical characterization of PP13

Dr. Bohn’s scientific vision combined with his thorough work using state-of-the-art methods of the 70’s and 80’s yielded the discovery of 26 soluble placental tissue proteins, among which PP13 was purified, physico-chemically characterized and described in 1983 (3). Dr. Bohn homogenized term placental tissues and fractionated the protein extracts by a step-wise process that included Rivanol and ammonium sulphate fractionation, gel-filtration, ethanol precipitation, and immunoabsorption techniques. The resulting PP13 protein was >99% pure, and SDS-polyacrylamide gel electrophoresis found it to be composed of two identical ~16 kDa subunits held together by disulphide bonds. The carbohydrate content of PP13 was found negligible, a feature that later became important in understanding its functions (2, 3). Utilizing the purified PP13-specific rabbit antiserum, an electroimmunoassay and an Ouchterlony’s gel-diffusion test found an average amount of 3.7 mg PP13 in human term placentas and detected PP13 solely in the placenta among fetal and adult tissues. The sensitivity of a radioimmunoassay (0.8 ng/ml) that utilized this rabbit antiserum was insufficient to detect PP13 in maternal and fetal serum or in amniotic fluid (2–4). Indeed, this is in accord with the 0.1–0.4 ng/ml concentration range of PP13 in maternal blood as was discovered with sandwich ELISA techniques using mouse monoclonal antibodies a decade later (5).

### Cloning, sequencing and initial molecular biological analysis of PP13

Professor Gábor N. Than’s team in Hungary isolated the full-length cDNA (GenBank Acc. No.: AF117383) encoding PP13 from a human placental cDNA expression library using Dr. Bohn’s rabbit anti-PP13 antiserum. Sequence analysis revealed a 578 bp insert with a 417 bp open reading frame encoding a 139 amino-acid protein (6). The predicted molecular mass and amino-acid composition of the cloned protein corresponded with Dr. Bohn’s estimate of the purified PP13 protein. A BLAST search of nucleotide and protein sequences showed PP13 to be homologous to members of the beta-galactoside binding galectin family, and computer analysis detected 8 out of 16 invariant residues in galectins conserved in PP13, suggesting its place in the galectin family (6). The highest sequence similarity of PP13 was found with the eosinophil Charcot–Leyden Crystal (CLC) protein, which forms crystals at sites of eosinophil-associated inflammation (7), a phenomenon similar to that found with PP13 immunostainings on first trimester decidual tissue sections (8). Interestingly, using a functional assay and highly sensitive NMR measurements, the native and recombinant PP13 was observed to have weak lysophopholipase activity (6, 9) similar to CLC protein. This lysophopholipase activity was also inferred from the release of free fatty acids from cultured primary trophoblasts exposed to PP13 (5). However, it was later revealed that this enzymatic activity of CLC protein is caused by an associated lysophospholipase (10), and further research is required to understand whether the lysophospholipase activity of PP13 is intrinsic or indeed related to an associated protein.

### Detailed structural and functional characterization of PP13 as a galectin

**Figure 2:**
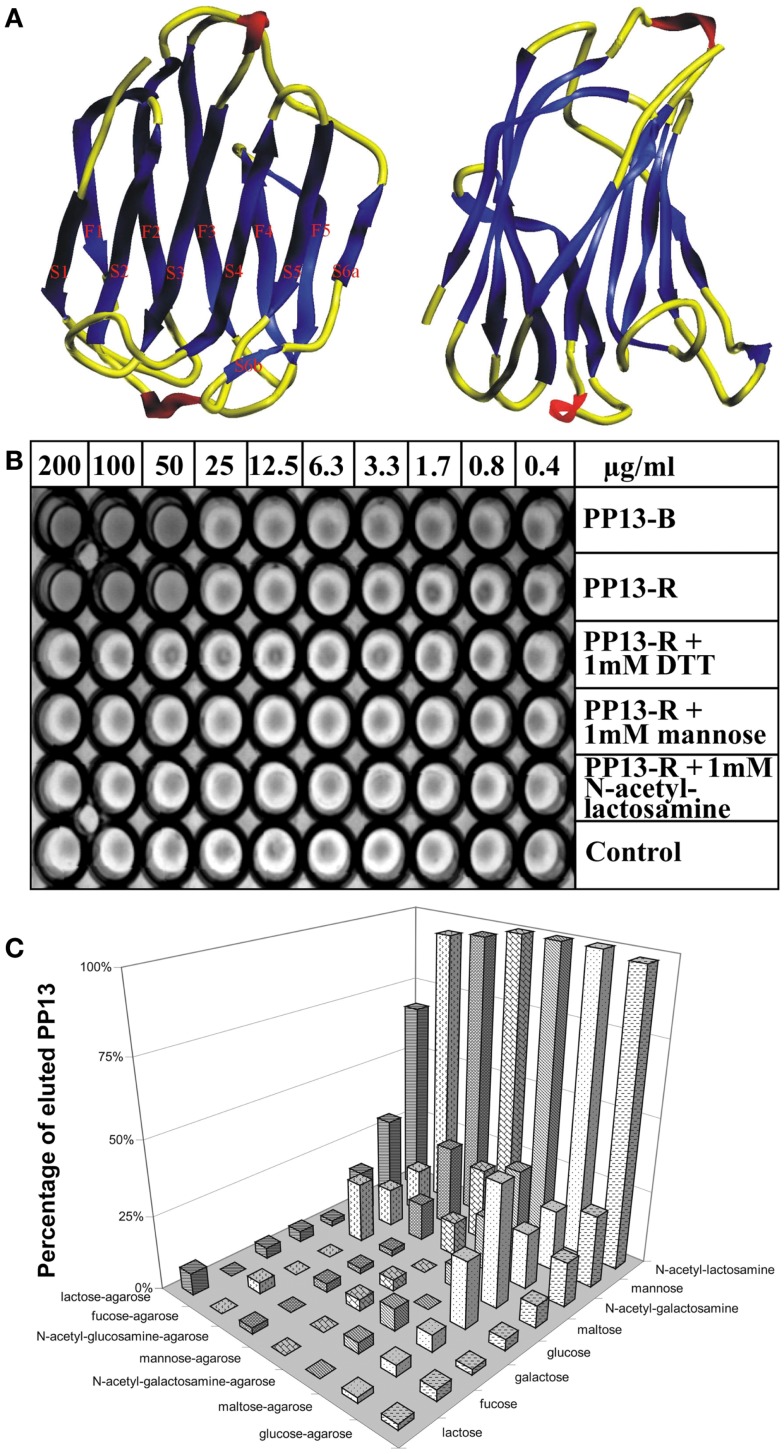
**The structural and functional basis for renaming of PP13 as galectin-13**. **(A)** The figure depicts the “jelly-roll” fold of PP13, which consists of five- and six-stranded β-sheets linked by two α-helices. **(B)** *In vitro* assays revealed haemagglutinating activity of placenta-purified (PP13-B) and recombinant (PP13-R) PP13. In non-reducing conditions, both PP13-B and PP13-R agglutinated human erythrocytes at ≥50 μg/ml protein concentrations. The haemagglutinating ability of PP13-R was inhibited by dithiothreitol or sugars at ≥1 mM concentrations. **(C)** The strength of PP13-R binding to sugar-coupled agarose beads increased from lactose-agarose to glucose-agarose (left to right). PP13 bound to sugar-coupled agarose beads was competitively eluted by sugars (1 M) listed back to front. PP13 had the best eluting capacity (sugar affinity) for *N*-acetyl-lactosamine, mannose and *N*-acetyl-galactosamine. Figure **(A)** was published in Ref. (11), and Figures **(B,C)** in Ref. (9). Kind permission for the reuse of figures was obtained from Oxford University Press **(A)** and John Wiley and Sons **(B,C)**.

The homology of PP13 to members of the galectin family inspired further structural and functional investigations. Homology modelling based on the “jelly-roll” fold of galectins observed by X-ray crystallography revealed the 3D model of PP13, which was deposited into the Protein Data Bank (Acc. No.: 1F87) (11). This fold consists of five- and six-stranded β-sheets linked by two α-helices characteristic for “prototype” galectins (Figure 2A). Out of the eight consensus residues in the galectin carbohydrate-recognition domains (CRDs), four identical and three conservatively substituted residues were found in PP13. Computational docking simulations showed that the PP13 CRD may bind sugars, e.g. *N*-acetyl-lactosamine and lactose, similar to most galectins (Figure 2A). Since these lines of evidence demonstrated that PP13 is a novel galectin, it was designated as galectin-13 (11).

Galectins constitute a subgroup among the superfamily of lectins, carbohydrate-binding proteins that are important in the regulation of cellular interactions with cells, the extracellular matrix and pathogens. They bind to glycans residing on glycoproteins, glycolipids and other glycoconjugates that constitute a complex array coined the “glycome”, which stores orders of magnitude larger biological information than nucleic acids and proteins store. For example, the numbers of 4,096 hexanucleotides and 64 million hexapeptides are far surpassed by the 1.44 × 10^15^ isomer quantity of hexasaccharides (12). Galectins can bind to a diverse set of glycoconjugates, and therefore, they have pleiotropic functions in a variety of key biological processes including signal transduction, cell differentiation, apoptosis, or cell adhesion. Moreover, galectins are positioned at the cross-roads of adaptive and innate immune functions as they are key determinants of acute and chronic inflammation, immune tolerance and host-pathogen interactions (12–18).

Triggered by the recognition that these galectin functions are important determinants of healthy pregnancies (19), the detailed molecular characterization of PP13 allowed greater insight into its function in the placenta during pregnancy. Similar to other galectins, PP13 also hemagglutinated human erythrocytes *in vitro* (Figure 2B). Furthermore, sugar-binding assays showed the affinity of PP13 for carbohydrates widely expressed in the human placenta (Figure 2C), particularly for *N*-acetyl-lactosamine, mannose and *N*-acetyl-galactosamine (9) as already predicted by molecular modelling (11). Assay performance in reducing conditions decreased the hemagglutinating (Figure 2B) and sugar binding activity of PP13, suggesting that homodimerization of PP13 subunits by disulphide bridges are important for these functions (9).

Through placental immunostaining, PP13 was found in the cytoplasm and brush border membrane of the syncytiotrophoblast. Using affinity chromatography and mass spectrometry, annexin II and beta/gamma actin were identified as ligands of PP13, a finding that was also supported by high colocalization of PP13 with annexin II in the syncytiotrophoblast brush border membrane. These results suggested the galectin-like externalization of PP13 to the cell surface by extracellular vesicles containing actin and annexin II (9). It has to be elucidated whether, similar to other galectins (20), PP13 may bind to glycoconjugates on cell surfaces and form “galectin-glycan lattices” that are important in cellular interactions and signaling.

### The Interactions of PP13 with ABO blood group antigens

**Figure 3:**
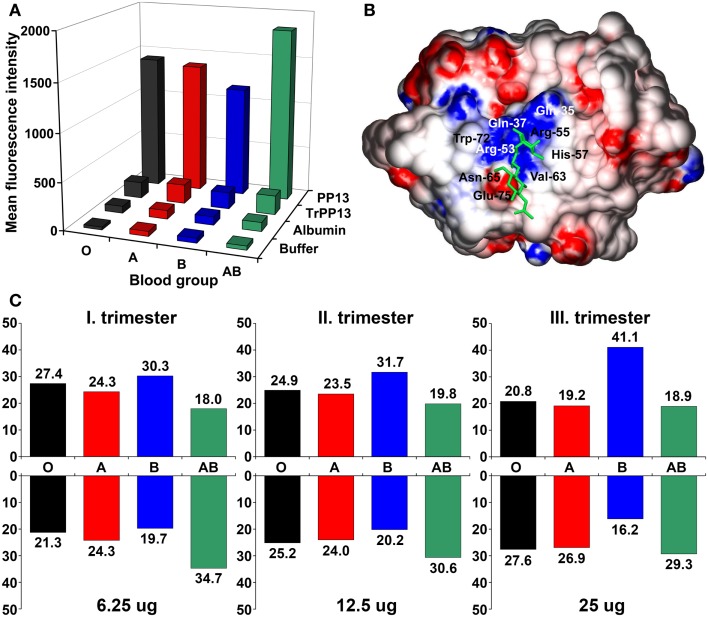
**The differential binding of PP13 to ABO blood group antigens *in vitro* and *in vivo***. **(A)** Flow cytometry showed that recombinant PP13 binding to erythrocytes was specific and mediated by its carbohydrate- recognition domain (CRD) since recombinant truncated PP13 (TrPP13) bound negligibly similar to bovine serum albumin (BSA). PP13 had the strongest affinity to blood group AB erythrocytes and weakest affinity to blood group B erythrocytes. **(B)** Surface representation of PP13 complexed with blood group H trisaccharide (green). Blue and red colors indicate positive and negative electrostatic potentials on the molecular surface, respectively. The binding groove of the CRD contains a central positive channel flanked by negative regions. **(C)** The relative binding of PP13 (lower panel) to different ABO blood group erythrocytes dynamically changed and inversely mirrored the relative serum PP13 concentrations (upper panel) in women with different ABO blood groups with advancing gestation from the first to third trimesters. All figures were published in Ref. (21). Kind permission for the reuse of figures was obtained from the Public Library of Science.

As an indirect sign of PP13 binding to glycoconjugates on cell surfaces, placental immunostainings showed PP13 positivity of maternal and fetal erythrocytes, confirming the *in vivo* erythrocyte-binding of PP13 (21). These results were consistent with the tendency of PP13, similar to other galectins, to bind beta-galactosides that are present at terminal positions on ABO blood-group antigens (9, 11, 22, 23). Flow cytometry measurements further demonstrated the binding of PP13 but not its CRD-truncated variant to erythrocytes, proving that PP13 binding is mediated by its CRD (Figure 3A). PP13 binding was similar in intensity to blood group A and O erythrocytes, while PP13 had the weakest binding intensity to blood group B and the strongest binding intensity to blood group AB erythrocytes (Figure 3A). Similar to other galectins (24, 25), PP13 binding to various ABO blood group erythrocytes changed dynamically with increasing PP13 concentrations (Figure 3C).

Computational studies have indicated that the structural basis for ABO blood group antigen binding includes the following: (1) three out of four residues in the core galectin CRD involved in disaccharide-binding are conserved in PP13 (11, 23); (2) PP13 has a similar “B-site” involved in ABO antigen binding as human galectins that exhibit ABO blood group antigen binding (21, 25); and (3) PP13 accommodates blood group H trisaccharide in its CRD similar to a fungal galectin’s CRD (21) (Figure 3B).

It is interesting to note that ABO blood group antigens are oligosaccharides attached to cell-surface glycoconjugates on epithelia, endothelia and erythrocytes, which might have been evolutionarily advantageous in conferring resistance against certain pathogens (26). The gene encoding for the enzymes that catalyze the transfer of these oligosaccharides to cell-surface glycoconjugates emerged in primates (22, 27). If the evolution of the ABO blood group system and genes encoding for PP13 and closely related galectins was somehow associated, that would suggest a potential functional relevance of PP13 binding to ABO blood group antigens.

## The Evolution and Human Disease-Related Polymorphisms of *LGALS13*

### Evolutionary analyses of genes encoding for PP13 and closely related galectins

An evolutionary study presented compelling evidence that a cluster of galectin genes, including *LGALS13* that encodes PP13, emerged on chromosome 19 in anthropoid primates, which differ from other primates by having larger brains and longer gestations (23). The analysis of this galectin cluster in the available genome assemblies revealed frequent gene duplication, inversion and deletion events characteristic of repeat-mediated “birth-and-death” evolution, a process that leads to novel phenotypes in species adapting to their changing environment (28). Detailed analysis showed that transposable long interspersed nuclear elements (LINEs) were positioned at the majority of boundaries of large inversions and gene duplication units, suggesting that LINEs had mediated the rearrangements within this cluster. Genes in this cluster have four-exon structures as other “prototype” galectin genes. Of the two major clades in the cluster, one contains genes with predominant placental expression including *LGALS13* and related pseudogenes. Of note, *LGALS13* was only found in Old World monkeys and apes. Sequence analyses of 24 newly determined sequences and 69 annotated sequences in 10 anthropoid species indicated functional diversification among PP13 and related galectins during evolution as can be inferred from the amino acid replacements in their CRDs (23).

**Figure 4:**
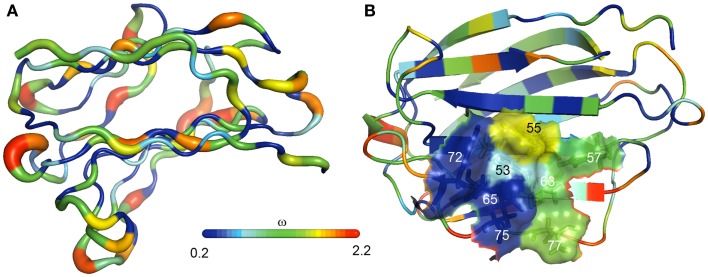
**The evolution of PP13 and closely related galectin genes in the chromosome 19 cluster**. **(A)** Evolutionary changes leading to structural diversification in chromosome 19 cluster galectins are depicted on the molecular backbone of PP13 (left). The width and color of the ribbon varies in proportion with site-specific ω values (d_N_/d_S_; ω < 1, purifying selection; ω > 1, positive selection) for chromosome 19 cluster galectins. ω, indicated by the color spectrum on the bar, is the smallest along β-strands and highest in loop regions. **(B)** The same color coding shows that four residues in the PP13 CRD (residues: 53, 65, 72, 75) have been conserved in chromosome 19 cluster galectins, while the other four residues on the opposite side of the CRD (residues: 55, 57, 63, 77) show more evolutionary changes among these galectins.

Sequence comparison showed a strong conservation of more than half of the residues of PP13 and cluster galectins predominantly located in the protein cores that determine their overall structure, whereas residues on their surface, especially in the loop regions, have undergone rapid evolution (23) (Figure 4A). From the eight conserved residues in the galectin CRD, four (positions: 53, 65, 72, and 75) that are key in the overall sugar binding form a pocket in one side of the CRD and were under purifying selection in PP13 and cluster galectins. The other four residues (positions: 55, 57, 63, and 77) on the opposite side of the CRD had more variability among cluster galectins, with frequent replacements in several lineages following gene duplications including K >T77 in PP13 (Figure 4B). As these latter four residues are crucial for galactose or glucose binding, the structural differences might have resulted in differing functions between PP13 and other cluster galectins. Indeed, functional experiments with human recombinant PP13 and five other galectins proved the different sugar-binding profiles of these investigated proteins (23).

A large number of pseudogenes in the studied species were found in the cluster (23). These emerged by the deletion of exons, mutations of the exon–intron boundaries, and by the introduction of one or more in-frame premature stop codons. As a striking observation, 18 out of the identified 38 pseudogene variants contained the “163C >T” DNA variant leading to the introduction of a premature stop codon at the site encoding residue 55, which may result in truncated proteins of 54 amino acids that lack the entire CRD. In fact, functional experiments proved that this “163C >T” DNA variant results in the expression of a truncated PP13 that cannot bind carbohydrates (23). The question why nature utilized the same process to silence so many galectin genes in certain lineages, including *LGALS13* in baboon, Bornean orangutan and Sumatran orangutan, remains unanswered.

### DNA variants in human *LGALS13*

Somewhat related to evolutionary selection, a total of 933 *LGALS13* DNA variants have been already identified in the genomes of 1,092 individuals from 14 populations by the “1000 Genomes Project”^1^. These included mostly upstream (*n* = 277), downstream (*n* = 261) or intron (*n* = 240) variants. Besides these, non-coding transcript (*n* = 98) or exon (*n* = 56) variants and missense variants (*n* = 56) were frequently detected. Nevertheless, the “1000 Genomes Project” has not provided information regarding the association of *LGALS13* DNA variants with disease susceptibility. In search of *LGALS13* DNA polymorphisms by targeted genotyping studies, the association of certain *LGALS13* DNA variants with severe complications of pregnancy has been identified. These studies utilized whole blood DNA samples obtained from pregnant women and their neonates in a South African cohort of the Black and Coloured population, and the following DNA variants were revealed for *LGALS13*: (1) variants in Exon 3 including single nucleotide polymorphisms (SNPs) and a single nucleotide deletion, which latter causes a frame-shift in the open reading frame, leading to the formation of a premature stop codon and a truncated protein (221delT); (2) SNPs in Introns 2 and 3 including an intron boundary polymorphism that is associated with alternative splicing and the deletion of Exon 2; and (3) an SNP in the promoter region (29–34).

Of interest, in a prospective cohort of 450 low-risk primigravid women of Black and Coloured origin, carrying the naturally occurring “221delT” mutation conferred a 2.27-fold relative risk for preterm labor (34). The frequency of heterozygous carriers of this mutation was higher in the group of women with preterm preeclampsia (5.7%) than in controls (2.4%), and no individuals were found to be homozygous. In another study conducted on the same population, there was a significant association for this mutation and preeclampsia, particularly among Coloured women (33). These results suggest that the placental expression of a functionally impaired, truncated PP13 may put women at increased risk for severe pregnancy complications. However, so far no polypeptide derived from the “221delT” DNA polymorphism could be identified in placental or body fluid samples, most likely due to the rapid degradation or insufficient immunodetection of such a protein because of the anticipated major misfolding (35).

The “-98A/C” promoter polymorphism was also associated with the risk of preeclampsia (31, 33, 34). In a prospective cohort of low-risk pregnant women, controls were in the Hardy-Weinberg equilibrium, while cases deviated from that, and the heterozygous A/C genotype appeared to be protective against preeclampsia (31). Another study comprising the same population found a significant difference between “-98A/C” genotype distributions in patients with placental abruption and controls among Coloured women (33). These results suggest that the “-98A/C” promoter polymorphism may negatively affect *LGALS13* expression and PP13 functions.

## The Expression Pattern of PP13 in Humans

### Wide-scale expression profiling of PP13 in human tissues

**Figure 5:**
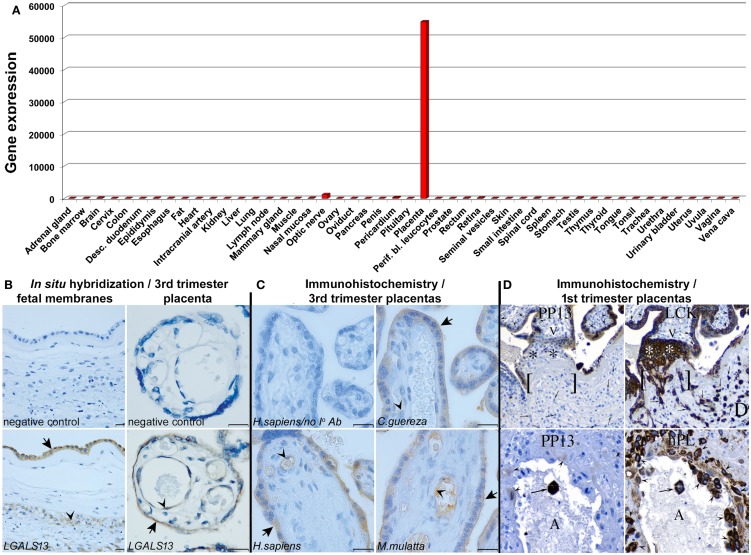
**PP13 expression profiling**. **(A)** qRT-PCR on a human 48-tissue cDNA panel uncovered that *LGALS13* is predominantly expressed by the placenta. The y axis shows gene expression levels [2^(−ΔCt)^]. **(B)** *In situ* hybridization revealed *LGALS13* expression in the amnion (arrow) and chorionic trophoblasts (arrowhead) in the fetal membranes in normal term pregnancy (left). In normal term placenta, *LGAL13* is predominantly expressed by the syncytiotrophoblast (arrow) and endothelium (arrowhead) (right). Scale bars: 20 μm. **(C)** PP13 immunostaining is conserved in the syncytiotrophoblast, its apical membrane (arrows), and the endothelia (arrowheads) of human and anthropoid primate placentas. (Scale bars: 20 μm.) **(D)** (Upper panel) Serial sections of a 15 week junctional complex stained for PP13 and low molecular weight cytokeratin (LCK). LCK immunostained epithelial cells, including cytotrophoblasts (arrowheads), anchoring trophoblasts (*), early infiltrating trophoblasts [ ], and invasive trophoblasts (arrows) in the decidua (D). Mesenchymal villous core cells (V) and decidual cells were negative. PP13 immunostaining was found in the syncytiotrophoblast. The cytotrophoblasts (arrowheads), anchoring trophoblasts (*), early infiltrating trophoblasts [ ], and the invasive trophoblasts (arrows) in the decidua (D) were negative. (Lower panel) Serial sections of 8 week maternal spiral arterioles immunostained for PP13 and human placental lactogen (hPL). All the decidual invasive, intravascular and endovascular trophoblasts (arrowheads), and a single luminal (A) syncytiotrophoblast (arrow) were stained for hPL. The monoclonal anti-PP13 antibody did not stain decidual invasive trophoblasts, lightly stained endovascular trophoblasts (arrowheads), and it intensely stained luminal syncytiotrophoblasts (arrow). Figure **(A)** represents data published in Ref. (23). Figures **(B,C)** were published in Ref. (23). Figure **(D)** was published in Ref. (8). Kind permission for the reuse of the figures was obtained from the National Academy of Sciences of the United States of America **(A-C)** and SAGE US **(D)**.

Besides the studies on *LGALS13* DNA variants, the investigations on the expression patterns of PP13 have revealed interesting insights. The study describing the cloning of PP13 also presented compelling evidence for the predominant placental expression of PP13 in the human body (6). In fact, the expression profiling of human adult and fetal, normal and tumorous tissues by Western blot (26 tissues) and Northern blot (16 tissues) detected a 16kDa PP13 immunopositive band in extracts of human term placentas, and unique placental PP13 mRNA expression, respectively. These findings were supported by GenBank evidence of only placental expressed sequence tags for *LGALS13*. Later, the wide-scale expression profiling of *LGALS13* and related chromosome 19 cluster galectin genes using qRT-PCR on a human 48-tissue cDNA panel confirmed the predominant placental expression of *LGALS13* (Figure 5A) (23).

### Placental expression profiling of PP13 in normal pregnancies

In human villous placental tissues at term, immunohistochemistry and immunofluorescence consistently found predominant PP13 positivity of the syncytiotrophoblast and villous capillary endothelium but not the cytotrophoblasts (8, 9, 21, 23, 36, 37). *In situ* hybridization (23) detected PP13 mRNA expression in the same placental cells, further confirming the specificity of the immunostainings (Figure 5B). The same PP13 expression pattern was found in the placentas of Old World monkeys, suggesting the conservation of PP13 expression during evolution (Figure 5C). Moreover, *in situ* hybridization revealed *LGALS13* expression in the amnion and chorionic trophoblasts in the fetal membranes. These findings showed PP13 expression predominantly in locations where maternal-fetal immune interactions occur.

In the first trimester, PP13 was immunolocalized to the syncytiotrophoblast and multinucleated luminal trophoblasts within converted decidual spiral arterioles (8). Villous cytotrophoblasts and invasive extravillous trophoblasts in the anchoring trophoblastic columns were immunonegative (Figure 5D). The syncytiotrophoblastic staining intensity declined with gestational age, being the strongest between 6 to 8 weeks of gestation. This study also confirmed previous findings in term placental tissues on predominant, diffuse cytoplasmic and also nuclear immunopositivity of the syncytiotrophoblast.

### Expression profiling of PP13 during villous trophoblast differentiation and fusion

Based on this immunohistochemical evidence, it was hypothesized that PP13 expression is related to the biochemical and morphological differentiation and syncytialization of the villous trophoblast (36). These processes are primarily governed by cyclic adenosine monophosphate (cAMP) and protein kinase A (PKA), which regulate the resetting of the transcriptional program during the shift from cytotrophoblast into the syncytiotrophoblast (38–40). The resulting unique transcriptome of the syncytiotrophoblast (41) controls the production of placental hormones, immune proteins and other proteins predominantly expressed by the placenta, which support pregnancy. Besides the exchange of feto-maternal gas, nutrients and waste, and the hormonal regulation of fetal development, the syncytiotrophoblast is also active in generating immune tolerance between the mother and her semi-allogeneic fetus (2, 42, 43).

*In vitro* assays with trophoblast-like BeWo cells demonstrated that indeed *LGALS13* expression is related to trophoblast fusion and syncytium formation induced by the cAMP-analogue Forskolin, and that a PKA inhibitor could block BeWo cell syncytialization and *LGALS13* expression (44). A recent study confirmed these findings in BeWo cells, and demonstrated the syncytialization and differentiation-related *LGALS13* expression in primary villous trophoblasts (45). The evolutionary and functional investigations of the trophoblastic regulatory mechanisms of *LGALS13* expression showed that promoter evolution and the insertion of an anthropoid-specific LINE element into the 5′ untranslated region (UTR) of an ancestral gene introduced binding sites for several transcription factors (e.g. ESRRG) key in villous trophoblastic gene expression, leading to the gain of placental expression of *LGALS13* and related chromosome 19 cluster galectin genes (43, 45). Glial cell missing-1 (GCM1), the transcription factor that governs villous trophoblast fusion and syncytialization (46), was shown to facilitate the expression of ESRRG and other key villous trophoblastic transcription factors, and thus, to indirectly promote the placental expression of *LGALS13* and cluster galectin genes. In addition, DNA methylation was also observed to regulate developmental expression of *LGALS13* and cluster galectin genes (45).

### Placental aspects of preeclampsia

It is important that the impairment of villous trophoblast syncytialization characterized by the decreased trophoblastic expression of GCM1 and syncytin-1, a fusogenic protein regulated by GCM1, has been observed in preeclampsia (47, 48), an obstetrical syndrome originating from impaired early placentation (49, 50). Preeclampsia is diagnosed by new-onset hypertension and proteinuria after 20 weeks of gestation, and it is a major cause of maternal, fetal and neonatal morbidity and mortality (51). Moreover, this syndrome consists of various subtypes defined by gestational age (e.g.: early-onset: <34th weeks; preterm: <37 weeks; and term: ≥37 weeks) (52, 53). Early-onset and preterm preeclampsia are severe subtypes of the disease that require premature delivery and are more often associated with intrauterine growth restriction (IUGR), hemolysis, elevated liver enzymes, and low platelet count (HELLP) syndrome, and fetal death, while term preeclampsia may be severe or mild in its clinical presentation (51–55). Although the molecular pathways of preeclampsia are incompletely understood, it appears to be associated with impaired placentation as the only definite therapy of preeclampsia is still the delivery of the fetus and the removal of the placenta (50, 51, 53). It is also evident that heterogeneous causes can trigger early placental pathologic events, and that these are followed by the onset of the terminal pathway of preeclampsia in a later stage, leading to the subsequent clinical onset of the symptoms (50, 51, 56).

Several studies providing histopathologic or transcriptomic evidence have suggested that the placental pathogenesis of preeclampsia may differ in its subtypes as more pronounced differences could be observed in early-onset than late-onset preeclampsia when compared to gestational age-matched controls (57–61). In line with these findings, the extent of histopathologic changes in the placental bed was most extensive in early-onset preeclampsia, especially in cases associated with IUGR. These abnormal findings were consistent with impaired trophoblast invasion into the uterine tissues and the consequent abnormal remodelling of the maternal spiral arterioles, placental pathologic events that occur in the first trimester (62, 63).

Previously it was thought that impaired early placentation is associated with placental hypoxia (64); however, it has recently become evident that the resulting fluctuation in uterine blood supply leads to placental ischemic injury, causing oxidative stress, pro-inflammatory conditions, and apoptosis (65–67). In response, the placenta expresses and releases increased amounts of anti-angiogenic factors, pro-inflammatory cytokines and aponecrotic syncytiotrophoblast microvesicles. The latter might induce maternal anti-angiogenic and exaggerated systemic pro-inflammatory states, hypertension and proteinuria (50, 51, 53, 66, 68–70).

### Decreased placental PP13 expression in preeclampsia

**Figure 6:**
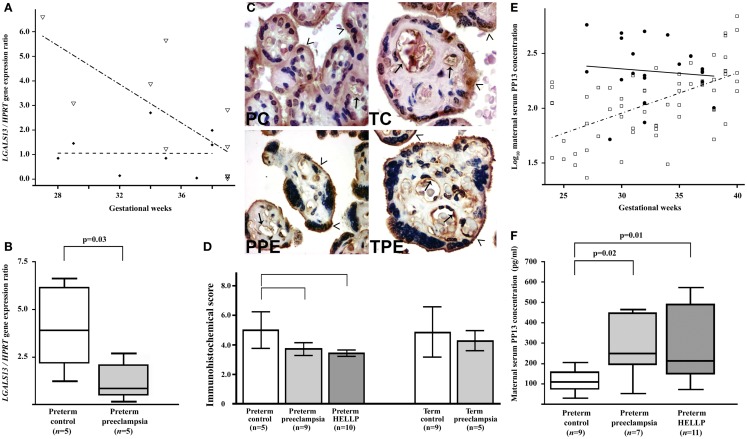
**Decreased placental *LGALS13* expression and increased trophoblastic PP13 shedding in preterm preeclampsia**. **(A)** Relative *LGALS13* expression decreases with advancing gestational age in controls (open triangles), while it is constantly low in patients with preeclampsia (diamonds). **(B)** Relative *LGALS13* expression is lower in women with preterm preeclampsia than in controls. **(C)** Syncytiotrophoblastic PP13 immunostaining is decreased in preterm preeclampsia compared to controls. PC: preterm control, 35 GW (weeks of gestation); TC: term control, GW38; PPE: preterm preeclampsia, GW29; TPE: term preeclampsia, GW37. The endothelium (arrows) is also PP13 immunopositive in all sections. The microvillous membrane (open arrowheads) stains moderately for PP13 in controls, while it is strongly PP13 immunopositive in preeclampsia. 500× (left) or 700× (right) magnification. **(D)** The immunohistochemical score of the syncytiotrophoblast is higher in preterm controls than in preterm preeclampsia, with or without HELLP syndrome, while it is not different between cases and controls at term. **(E)** Maternal serum log_10_ PP13 concentrations increase as a function of gestational age in control women (open rectangle), while these do not correlate with gestational age in patients with preeclampsia (filled circle). The regression line for log_10_ PP13 concentrations is significantly different in the two groups. **(F)** Median maternal serum PP13 concentrations are higher in women with preterm preeclampsia, with or without HELLP syndrome, than in controls. All the figures were published in Ref. (36). Kind permission for the reuse of figures was obtained from Springer Science+Business Media.

In this context, *LGALS13* expression was found to be down-regulated in villous placental tissues in preeclampsia. This was first described for preterm preeclampsia compared to gestational age-matched controls at the time of disease, and this phenomenon was suggested to be associated with problems in trophoblast syncytialization (36) (Figures 6A,B). Since then, other studies confirmed these findings in the third trimester, including one that investigated placental *LGALS13* expression at the time of disease (71) and another that looked for syncytiotrophoblastic *LGALS13* expression in laser captured specimens in the first trimester (72). The latter study detected decreased *LGALS13* expression in the syncytiotrophoblast from chorionic villus samples obtained at 11 weeks of gestation in women who subsequently developed preeclampsia compared to controls. Such first trimester down-regulation of placental *LGALS13* expression may be one of the earliest pathological indications for the subsequent development of preeclampsia.

A recent study has revealed the possible molecular mechanisms leading to decreased placental *LGALS13* expression in women with severe preterm preeclampsia. It was found that in this subform of preeclampsia there is a decreased placental expression of *GCM1* and *ESRRG*, genes encoding transcription factors that regulate trophoblastic *LGALS13* expression (45). Moreover, functional experiments showed that the knock-down of *GCM1* in BeWo cells led to the down-regulation of ESRRG and other transcription factors that regulate *LGALS13* expression. Accordingly, it was concluded that there is a decreased GCM1-mediated trophoblast fusion and trophoblastic gene expression in severe preterm preeclampsia that leads to the down-regulation of *LGALS13*. Furthermore, the differential methylation of *LGALS13* was also found in the villous trophoblast in preterm preeclampsia, which may interfere with *LGALS13* expression, suggesting that potential additional disease-mechanisms may account for the trophoblastic pathology in preterm preeclampsia (45).

### Altered placental localization and increased shedding of PP13 in preeclampsia

**Figure 7:**
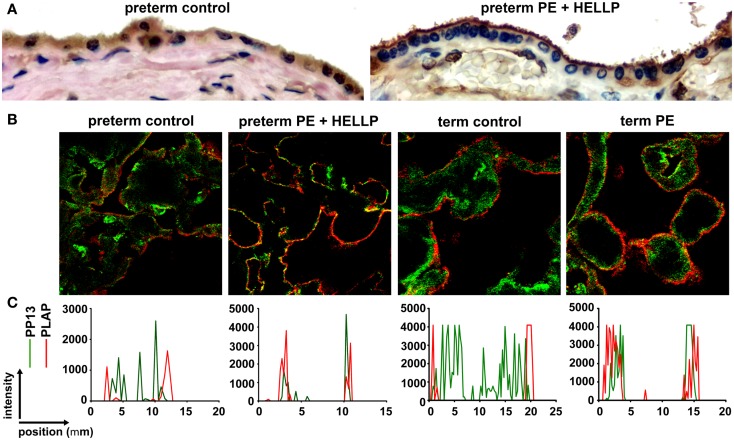
**Subcellular relocalization of PP13 in preeclampsia and HELLP syndrome**. **(A)** Representative images show uniformly moderate cytoplasmic and brush border membrane PP13 immunostaining of the syncytiotrophoblast in a preterm control placenta (left), while its weak cytoplasmic and strong membrane immunostaining in a placenta from a woman with preterm preeclampsia (PE) associated with HELLP syndrome (right). Cytoplasm protrusions, membrane blebs and shed membrane microvesicles immunostained intensely for PP13 (right). 800 x magnification. **(B)** Representative confocal images show the subcellular relocalization of PP13 (green) near to placental alkaline phosphatase (PLAP) immunopositive (red) lipid rafts in the juxtamembrane regions of the syncytiotrophoblast in term preeclampsia and preterm preeclampsia associated with HELLP syndrome compared to controls. **(C)** Line scan intensity distributions of PP13 and PLAP in representative confocal images shown in subfigure **(B)**. Figure **(A)** was published in Ref. (36). Figures **(B,C)** were published in Ref. (73). Kind permission for the reuse of the figures was obtained from Springer Science+Business Media **(A)** and Elsevier **(B,C)**.

In accord with gene expression data, immunostainings revealed that cytoplasmic PP13 positivity of the syncytiotrophoblast was weaker in preeclampsia compared to controls, particularly in preterm cases. Similar changes were also observed at the time of disease in preterm HELLP syndrome (36) (Figures 6C,D). Paradoxically, PP13 immunostaining of the syncytiotrophoblast microvillous membrane was stronger in preeclampsia and HELLP syndrome compared to controls (Figures 6C, 7A). Syncytial cytoplasmic protrusions and membrane microvesicles shed from the syncytiotrophoblast stained strongly for PP13 in preeclampsia (Figure 7A). It was suggested that the increased release of PP13 positive microvesicles from the syncytiotrophoblast may lead to elevated maternal serum PP13 concentrations in preterm preeclampsia and HELLP syndrome before or at the time when the clinical symptoms of preeclampsia appear (Figures 6E,F) (36, 37).

**Figure 8:**
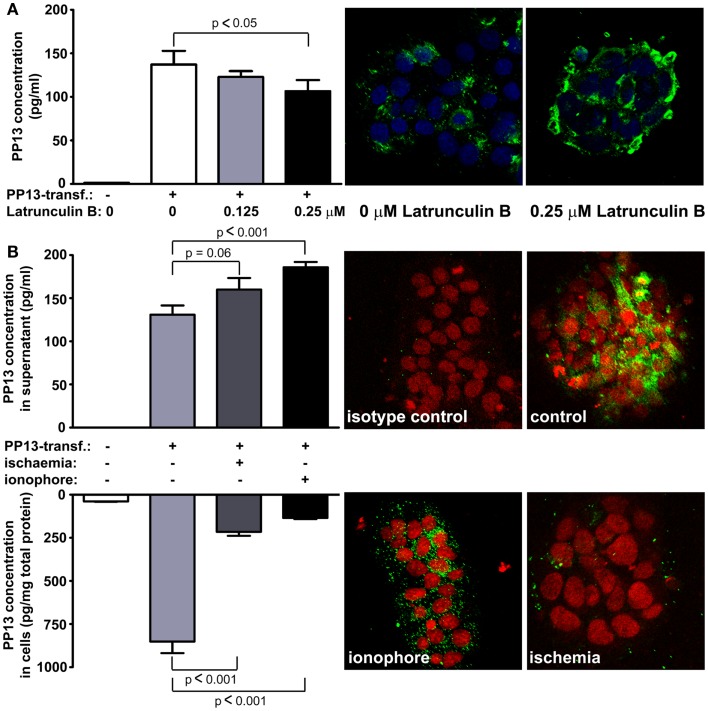
**Blocking of actin polymerization inhibits while calcium and ischemia promotes trophoblastic PP13 release**. **(A)** PP13 content of BeWo cell culture supernatants of non-transfected controls, as well as *LGALS13*-transfected, untreated or Latrunculin B-treated cells were measured by ELISA (left). *LGALS13*-transfected, untreated or Latrunculin B-treated cells were stained with anti-PP13 (green) and nuclei were counterstained with DRAQ5 (blue) followed by confocal microscopic analysis (right; 40x magnifications). The disruption of the actin cytoskeleton with Latrunculin B treatment decreased PP13 release from BeWo cells. **(B)** *LGALS13*-transfected BeWo cells were treated with calcium ionophore to increase intracellular calcium level, or kept under ischemic stress to mimic placental milieu in preterm preeclampsia. (Left) PP13 content of BeWo cells and cell culture supernatants of non-transfected controls, as well as *LGALS13*-transfected, untreated, calcium ionophore-treated or ischemia exposed cells were measured by ELISA. (Left) Representative confocal images of *LGALS13*-transfected control, calcium ionophore-treated or ischemia exposed BeWo cells immunostained with monoclonal anti-PP13 antibody (green) and counterstained with DRAQ5 (red) nuclei dye. Either ionophore treatment or ischemia induced the release of PP13 from BeWo cells. Figures were published in Ref. (73). Kind permission for the reuse and modification of the figures was obtained from Elsevier.

The subcellular redistribution of PP13 in the syncytiotrophoblast was further observed by confocal imaging of placental samples from patients with preeclampsia and HELLP syndrome compared to gestational age-matched controls (73). In all study groups, PP13 highly colocalized with placental alkaline phosphatase, a glycophosphatidylinositol-anchored lipid raft-resident protein. However, there was also a high degree of colocalization of PP13 with CD71, a non-raft plasma membrane protein, which decreased in preterm preeclampsia and HELLP syndrome. In contrast, the colocalization of PP13 with cytoskeletal actin, a protein earlier found to bind to PP13 with high affinity (9), was increased in all patient groups compared to controls. These results indicated that the translocation of PP13 to the juxta-membrane region of the syncytiotrophoblast in preeclampsia and HELLP syndrome is associated with actin (Figures 7B,C) (73). Supporting these observations in the placenta, subsequent *in vitro* experiments revealed that Latrunculin B, a selective blocker of actin polymerization, decreased PP13 release from BeWo cells and led to its intracellular accumulation (Figure 8A) (73).

This result may also explain how PP13 is released from the syncytiotrophoblast since the actin cytoskeleton and associated motor proteins drive intracellular and plasma membrane trafficking amongst a wide variety of cellular processes (74, 75). In this regard, galectins predominantly utilize unconventional trafficking routes, either vesicular or direct translocational, avoiding the endoplasmic reticulum (ER) and Golgi apparatus, since they are synthetized on free ribosomes and lack an *N*-terminal signal sequence for the translocation to the ER/Golgi system (76–78). However, other vesicular transport mechanisms for PP13 cannot be excluded, such as the “kiss and run” exocytosis, which was described for many hormones and neurotransmitters and was proved to be an actin- and calcium-dependent process (79, 80).

The role of actin cytoskeleton in the release of extracellular vesicles (EV; e.g. microvesicles/microparticles and exosomes), which also carry various galectins, has also been demonstrated (81–83). In addition, annexin II, another protein that specifically bound to PP13 (9), has also been found in various types of EVs along with actin (84, 85). Similar to galectin-9, which was shown to be associated with many different types of EV fractions (86), PP13 may also translocate with different EVs through the syncytiotrophoblast membrane. This type of release is supported by evidence on the PP13 release from BeWo cells mediated by exosomes (73) and the observed PP13 immunopositivity of microvillous membrane microvesicles shed from the syncytiotrophoblast (36).

### The Role of calcium and ischemia in trophoblastic PP13 release

Recent *in vitro* experiments with BeWo cells transfected with *LGALS13* to enable high PP13 expression (73) also showed an increased PP13 release from calcium ionophore-treated cells, evidenced by decreased cellular PP13 content and elevated amounts of PP13 in cell culture supernatants (Figure 8B). This finding is in accord with a previous report showing that galectin-3 is secreted by exosomes from monocytes upon calcium ionophore treatment (87). Calcium serves as a ubiquitous second messenger responsible for controlling numerous cellular processes including exosome secretion (88). Since calcium regulates the actin cytoskeleton at multiple levels including the organization of actin monomers into actin polymers and the super-organization of actin polymers into a filamentous network (89), it is not surprising that stimuli resulting in the elevation of intracellular calcium concentration can induce microvesiculation and membrane shedding of exposed cells (90, 91). As a mechanism, the dynamics of actin assembly and disassembly is regulated by certain actin-binding proteins such as annexin II in a calcium-dependent manner (92–96).

The release of PP13 from BeWo cells appears to be similar to the *in vivo* release when comparing the effect of calcium ionophores and ischemic stress (Figure 8B) (73). Ischemic stress of the placenta is a major component of the pathophysiology of preterm preeclampsia (97). In accord, higher PP13 release was observed in placental villous tissue explants obtained from women with preeclampsia compared to gestational age-matched controls in the third trimester (98). A possible explanation is that ischemic stress causes elevation in intracellular calcium levels, which leads to actin depolymerization supported by findings of separate studies (99, 100). All of these results indicate that different kind of actin- and calcium-dependent release mechanisms exist side by side for PP13, and most probably the dominant sort depends on the cell type and also on the nature of the received stimuli.

As a functional aspect of the increased PP13 release from the placenta in preeclampsia, ischemic and other stress conditions pose danger to the organism, which is signaled to the immune system by endogenous danger signals called “alarmins” (101, 102). Indeed, “danger signals” in the placenta have also been proposed to create an abnormal placental cytokine milieu and link the activation of the innate immune system and preeclampsia (8, 103–105). In this context, some galectins with cytokine-like properties (106, 107) may act as alarmins, since they are increasingly secreted from inflamed or damaged tissues, and they may elicit effector responses from innate and adaptive immune cells (19, 102, 108). Although direct evidence for the role of PP13 as an alarmin has not yet been established, these findings suggest that PP13 may function in such way in the placenta in preeclampsia (19, 73).

## *In vitro* and *In vivo* Functional Studies on PP13

### *In vitro* paracrine effects of PP13 on human immune cells

**Figure 9:**
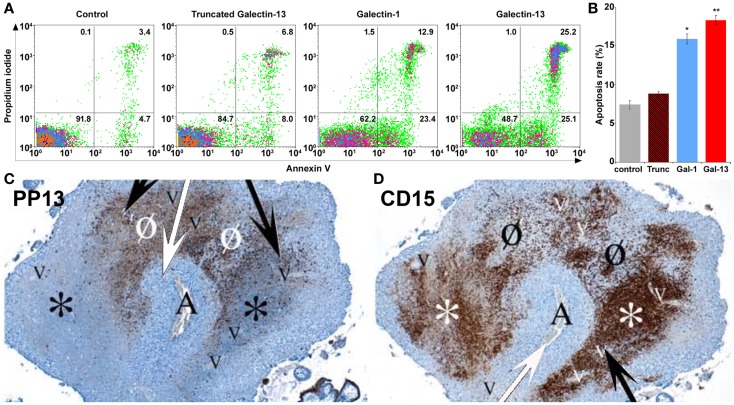
**Extracellular PP13 induces apoptosis *in vitro* and *in vivo***. **(A)** The *in vitro* apoptosis-inducing effect of PP13 on activated CD3+ T cells was comparable or stronger than that of galectin-1, whereas truncated PP13 did not have such effect when proteins were applied in 8μM concentration. Numbers in quadrants indicate the percentage of CD3+ T cells. **(B)** The *in vitro* apoptosis-inducing effect of PP13 on activated CD3+ T cells was stronger than that of galectin-1. Apoptosis rate was calculated as the percentage of Annexin V and propidium iodide double-positive cells. Gal: galectin; Trunc: truncated galectin-13; *P < 0.05; **P < 0.01. **(C,D)** Serial sections of decidua basalis samples in the first trimester. A spiral arteriole (“A” and white arrows) is surrounded by decidual veins (“V” and black arrows). **(C)** PP13 immunostainings revealed areas of intense PP13 depositions consistent with early and active zones of necrosis (“ZONE”) formation (Ø), and areas with weak PP13 immunoreactivity consistent with end-stage “ZONEs” (*). **(D)** CD15 immunostainings revealed neutrophil accumulation showing an inverse pattern with PP13 depositions. The least intense staining was observed in early “ZONEs” (Ø), while the most intense staining in end-stage “ZONEs” (*). Figures and data **(A,B)** were published in Ref. (23). Figures **(C,D)** were published in Ref. (8). Kind permission for the reuse and modification of figures was obtained from the National Academy of Sciences of the United States of America **(A,B)** and SAGE US **(C,D)**.

PP13 released from the trophoblast into the extracellular space may have various functions similar to other galectins, which may exert their pleiotropic extracellular functions in an autocrine and paracrine manner. Since PP13 is secreted by the trophoblast to the maternal circulation from where it gets into the decidual extracellular matrix (8), PP13 may affect various types of circulating and tissue-resident maternal leukocytes throughout pregnancy. Thus far only a couple of functional experiments were carried out, focusing on the examination of potential extracellular effects of PP13. As several members of the galectin family regulate adaptive immune responses by the induction of apoptosis of activated T lymphocytes (19, 109–111), the apoptosis-inducing effects of PP13 and other chromosome 19 cluster galectins were investigated on activated T cells freshly isolated from healthy donors (23). Among the studied recombinant galectins, PP13 had the strongest apoptosis-inducing effect (Figures 9A,B), stronger than galectin-1, a protein that has central role in maintaining maternal-fetal immune tolerance in eutherian mammals (16, 19, 112). A subsequent study also investigated the effect of PP13 on the secretion of cytokines and chemokines from mononuclear cells isolated from peripheral blood of pregnant women (8). The treatment with placenta-purified PP13 slightly increased the secretion of interleukin (IL)-1α and IL-6 into the culture medium. These *in vitro* experimental evidences suggest various effects of PP13 on immune cells, which may also be largely dependent on the type, activation and differentiation status of the affected cells, the microvesicle-bound or free nature and concentration of PP13, and the redox status of the environment, similarly to other galectins (19).

As with galectins that bind ABO blood group antigens, the paracrine effects of PP13 may also be affected by this phenomenon (21). In this context, large cohort studies showed that preeclampsia is more frequent among patients with AB blood group compared to those with non-AB blood groups (113, 114). Thus, recently it has been hypothesized that the higher susceptibility to preeclampsia among AB blood group women may be related to the decreased bioavailability and paracrine effects of PP13 on maternal immune cells (21). As a similar phenomenon, the ABO blood group antigens linked to the protein backbone of coagulation factor VIII and von Willebrand factor significantly affect the bioavailability of these blood clotting factors and coagulation (115–118). These findings altogether further underline the important immunoregulatory functions that PP13 may have in early pregnancy and warrant further investigation of the effect of ABO blood group system on PP13 bioavailability and functions. In summary of all of the above, PP13 may have a complex role in the regulation of adaptive and innate immune functions at the maternal-fetal interface depending on the changing environment.

### The *In vivo* paracrine effects of PP13 on human immune cells

This latter study also described an interesting finding in placental tissue specimens obtained from elective terminations of pregnancies between 6 to 15 weeks of gestation (8). In addition to the PP13 immunopositivity of the syncytiotrophoblast, crystal-like PP13 deposits in the decidual extracellular matrix and phagocytosed PP13 immunopositive material in immune cells were also documented. These deposits were always adjacent to decidual veins but not to arteries, and they were coincident with unique zones of necrotic and apoptotic immune cells (“ZONEs”) (Figures 9C,D) (8), a phenomenon that may be consistent with previous findings on zones of decidual necrosis in the first trimester (119). In addition, immunostainings demonstrated the expression of IL-1α and IL-6 within and around macrophages in these “ZONEs”, suggesting the potential pro-inflammatory effect of PP13 (8). The highest number of “ZONEs” appeared to be between 7 to 8 weeks of gestation, and their occurrence declined by the end of the first trimester in parallel with the completion of the spiral artery remodelling and the establishment of the blood circulation to the placenta. This study also revealed that spiral artery transformation by invasive trophoblasts and “ZONEs” were rarely seen in specimens obtained from women with low maternal serum PP13 concentration compared to those with normal PP13 values. In summary, these findings prompted the authors to suggest that syncytiotrophoblast-secreted PP13 reaches the decidual veins, crosses their wall, deposits into the extracellular matrix and forms perivenous crystal-like aggregates near the veins. The lysophospholipase activity associated with PP13 was implicated in this process, but no evidence yet exists to prove it. These PP13 deposits were suggested to serve as “diversion sites” to attract, activate and kill maternal immune cells, drawing these away from sites where the semi-allogeneic fetal trophoblasts invade and remodel maternal spiral arteries. It was also hypothesized that decreased PP13 expression may lead to deficient “ZONE” formation, decreased trophoblast invasion, and the subsequent failure of spiral artery transformation (8). Functional and causal evidence for these *in situ* observations needs to be provided in the future.

### PP13 and unique aspects of deep placentation in anthropoid primates

These *in vivo* findings are important from an evolutionary point of view since PP13 evolved in Old World monkeys and apes (23), species that have endovascular trophoblast invasion and spiral artery remodelling different from lower primates (120–123). In fact, a growing body of evidence suggests that PP13 may belong to primate-specific molecules (e.g. human leukocyte antigen C, killer-cell immunoglobulin-like receptors), which are involved in the regulation of immune mechanisms related to invasive placentation (124). The findings on PP13 and “ZONE” formation may be mostly related to the pro-apoptotic effect of PP13, similar to the effect of galectin-1 on activated decidual T cells, which is critical in the down-regulation of maternal adaptive immune responses at the maternal-fetal interface in early pregnancy (111). However, the pro-inflammatory action of extracellular PP13 may also fit with early placentation events.

In fact, the early pregnancy decidua is infiltrated by a large number of leukocytes, mainly natural killer (NK) cells (70%), macrophages (20–25%), and T cells (10%) (125–127). These leukocytes, especially decidual natural killer (dNK) cells, macrophages and T regulatory cells, are indispensable for the success of pregnancy since they produce a large variety of chemokines, cytokines, matrix metalloproteinases and angiogenic molecules that regulate maternal-fetal interactions, trophoblast invasion and spiral artery remodelling (127–130). On one hand, these immune cells are involved in the establishment of a delicate immune tolerance between the mother and the fetus, and on the other hand they promote local pro-inflammatory responses that facilitate implantation, trophoblast invasion and placentation events (126, 127). These complex immune interactions between the mother and the fetus are conveyed by cell membrane- and vesicle-bound as well as soluble molecules. Among the best studied molecular mechanisms are the effect of progesterone-induced blocking factor (PIBF) on the shift towards Th2 over Th1 cytokine production (131), the anti-inflammatory role of decidual macrophages (132), the immunosuppressive effects of decidual galectin-1 (16), trophoblastic indoleamine 2,3-dyoxignease (IDO), FAS/FAS ligand and galectin-1 (133, 134), and the roles of human leukocyte antigen (HLA)-C and HLA-G in protecting fetal cells from NK- and cytotoxic lymphocyte (CTL)-mediated cytolysis (135, 136). It is a question for future studies how the actions of PP13 are related to this complex, dynamically changing cellular and molecular network during placentation.

As the result of these complex interactions at the maternal-fetal interface, aggregates of extravillous endovascular trophoblasts plug the openings of uterine spiral arteries; therefore, they inhibit intervillous circulation at the beginning of gestation (49, 120, 128). This is suggested to be a protective mechanism to keep the developing embryo in a relatively low oxygen environment, minimizing oxidative stress that would lead to developmental defects during organogenesis (137). Of importance, the observed “ZONE” formation peaks when placental circulation is not yet established (8), and the low flow of endometrial gland secretions around the placenta allows the increased transport of PP13 from decidual veins into the decidua. This is also the period when extravillous trophoblast invasion starts into the decidua (128, 137). Remarkably, after the start of the placental intervillous circulation at around 8–10 weeks of gestation (49, 128, 137, 138) PP13 deposits and “ZONE” formation rapidly declines and diminishes by the time intervillous circulation is fully established at about 10–14 weeks of gestation (8). This suggests that PP13 transport is reduced to the decidual extracellular matrix due to the continuously increasing blood flow in spite of the increasing total production of PP13 by the placenta. Importantly, if trophoblastic plug formation is incomplete, placental circulation starts earlier, which leads to the oxidative stress of the placenta, the subsequent development of preeclampsia, and early pregnancy loss in more severe cases (49, 137, 138). In this context, the earlier start of placental blood flow would theoretically restrict PP13 transport into the decidua and “ZONE” formation, providing another mechanism to hamper normal placentation.

### The *In vitro* autocrine effects of PP13 on human trophoblast

An *in vitro* study showed the autocrine effect of PP13 measured by its ability to depolarize the membrane of primary trophoblasts isolated from normal and preeclamptic placentas (5). For these experiments, either the patch-clamp technique or a voltage-sensitive fluorescence dye was used, and PP13 was transiently added to the cells. PP13-induced trophoblastic membrane depolarization was increased with extracellular calcium concentrations according to the Nernst equation, and it was blocked in the presence of EGTA, a calcium chelator (5). Furthermore, a two-minute exposure of cells to PP13 resulted in linoleic acid release and subsequent prostacyclin liberation in a calcium-dependent manner. Galectin-1 did not elicit a similar response, indicating the specific effect of PP13. It is interesting that, in contrast, galectin-1 has various effects on trophoblasts including the regulation of hCG and progesterone production (139), proliferation (140), and syncytium formation (141). Based on these results, it would be interesting to further investigate additional autocrine signaling effects of PP13 on the trophoblast at various stages of syncytialization.

### The *In vivo* paracrine effects of PP13 in pregnant animals

Besides *in vitro* experiments, the *in vivo* effects of PP13 in an animal model have also been investigated. Initially, non-pregnant rats were exposed to a single bolus of intravenous PP13 injection followed by immediate hypotension and heart rate increase resulting from generalized vasodilatation (142). PP13 was also administered to pregnant rats subcutaneously via osmotic pumps that slowly released PP13 over a period of five days starting from day 15 of pregnancy. In these animals, the hypotension and increased heart rate lasted through the five days of PP13 administration. Furthermore, isolated uterine and mesenteric arteries responded with dilatation to *in vitro* PP13 treatment as measured by angiography (142). In subsequent studies, the effect of PP13 on uterine vasculature was investigated between days 8 and 15 of pregnancy during a prolonged intraperitoneal exposure through a slow release from osmotic pumps (35, 143). Again, PP13 treatment led to a general hypotension that lasted throughout the treatment period, and then blood pressures returned to normal. PP13 treatment affected uterine vasculature with the main effect elicited on uterine veins. These veins had an increased diameter on day 15, while their size returned to normal by day 20. Interestingly, PP13-treated rats delivered slightly larger pups and placentas than saline-treated controls, possibly because of the increased uterine blood flow in these animals (143). These findings may be related to the *in vitro* prostacyclin liberalization ability of PP13 (5).

PP13 is a primate-specific protein, and thus, certain differences exist between the set of potential “receptors” to which PP13 may bind in rats and primates. Moreover, various differences exist between primate and rodent gestations regarding the length of gestation, uterine anatomy, placentation, litter size, immune regulation, and other aspects. Therefore, the most appropriate context for the *in vivo* investigations of PP13 effects would be in a pregnant primate model; however, there are ethical limitations for such studies. While there could be major differences between the effects of PP13 in rats compared to humans due to the reasons described above, the effect of PP13 on hypotension and vasodilatation are novel and have not previously been described in regard to any other galectins. In the future, these *in vivo* effects of PP13 need to be further investigated in humans, presumably on placental bed arteries in hysterectomy specimens and also on placental derived *in vitro* decidual models in order to evaluate the potential therapeutic use of PP13 to prevent preeclampsia, along with many additional considerations.

### Functional considerations regarding the PP13 CRD

*In vitro* experiments on activated T cells also included their treatment with a truncated, 54-residue PP13 variant that lacks the entire CRD. This truncated protein was expressed from a mutated cDNA that contains the “163C >T” DNA variant frequently observed in cluster galectin pseudogenes in primates (23). Compared to the strong apoptosis inducing effect of PP13, this truncated PP13 had no effect on T cell apoptosis, confirming the crucial role of the CRD in this function (23). In addition, *in vivo* experiments included the administration of a different truncated PP13 (35), which contains the first 73 amino acids of PP13 similar to the “221delT” native mutant (34). Although this truncated PP13 variant contains 6 out of 8 amino acids from the CRD, its *in vivo* functional properties were different from PP13 since it caused hypotension in pregnant animals throughout the period of its active release between days 8 to 15 of pregnancy, while it did not increase the birth weight of the pups. Since an increased misfolding of this truncated protein was observed during the isolation from bacteria and the monoclonal anti-PP13 antibodies could not recognize it, it was concluded that its functional properties are different from those of the full length PP13 because of the misfolded structure (35). Further studies are warranted with these truncated PP13 variants to reveal their structural characteristics and effects.

## The Evaluation of PP13 in the Diagnosis of Preeclampsia

### Low circulating PP13 mRNA in maternal blood in preeclampsia

The discovery of fetal DNA and RNA in maternal blood stimulated the experimental assessment of free and cellular PP13 mRNA species in pregnant women’s blood in the first half of pregnancy. In accord with the previously discussed placental *LGALS13* expression data in preeclampsia, recent studies showed a lower PP13 mRNA content in the maternal blood in the first half of pregnancy in preeclampsia compared to matched controls (144, 145). The source of these PP13 mRNA species in maternal blood is only the placenta since no other human tissue expresses PP13 (23), and PP13 mRNA is not detectable in the blood of non-pregnant controls (146). These findings combined with those from placental studies have indicated that pathophysiological changes in PP13 expression appear very early in pregnancy. However, the predictive value of PP13 mRNA species in maternal blood is currently limited, which is most likely associated with varying and low amounts of trophoblastic mRNA reaching the maternal circulation. It is possible that advanced RNA processing techniques and sensitive detection methods like deep sequencing may enable a more robust PP13 mRNA detection in maternal blood for a better performance in preeclampsia prediction in early pregnancy. This aim is currently being supported by the European Union FP7 funded “ASPRE” project.

### First trimester maternal blood PP13 for predicting the risk of the development of preeclampsia

The evaluation of PP13 as a protein biomarker for the first trimester prediction of preeclampsia was analyzed with a recent meta-analysis based on studies performed with two immunoassay platforms (147). This meta-analysis explored 68 studies and included 19 into the final analysis, which were published between 2006 and 2013 (21, 37, 148–164). The analysis pooled the results from only singleton pregnancies of low or high risk women or all-comer cohorts, which were included in prospective or nested case-control studies, or fully prospective studies. A total of 16,153 pregnant women were tested for PP13 in the first trimester (between gestational weeks 6 and 14), among whom 1,197 developed subsequently preeclampsia. Out of these cases there were 19% who developed early-onset preeclampsia (<34 weeks) and 45% who developed preterm preeclampsia (<37 weeks).

Ten studies used the ELISA platform developed in Israel (21, 37, 148–152, 154, 160, 164), one study used the ELISA platform recently developed in China (161), and the remaining studies used the DELFIA platform. In all studies, PP13 blood concentrations were converted into gestational week-specific multiples of the medians (MoMs) (165), and then were further adjusted to maternal weight in two studies (156, 164) or to body mass index (BMI). In 10 studies, the PP13 MoMs were further adjusted to smoking, ethnicity, maternal age and parity. Interestingly, one study also adjusted PP13 MoMs to conception by *in vitro* fertilization (IVF) (164), and another study, which yielded the highest sensitivity and specificity, further adjusted PP13 MoMs to ABO blood groups (21).

All studies in the meta-analysis utilized algorithms that calculated the receiver operating characteristics (ROC) curves to detect the sensitivity and specificity, and logistic regression analysis to predict the risk of preeclampsia (147). When all cases of preeclampsia were included in the meta-analysis, the mean detection rate (DR) for predicting preeclampsia was 47% (95% confidence interval, CI: 43–65) at a 10% false positive rate (FPR). The DR of PP13 for preterm preeclampsia was higher, 66% (95% CI: 48–78), and for early-onset preeclampsia it was 83% (95% CI: 25–100). The assessment of likelihood ratios (LRs) for all cases of preeclampsia revealed a positive LR [sensitivity/(1-specificity)] of 5.82, a negative LR [(1-sensitivity)/specificity] of 0.46 and an overall LR (positive LR/negative LR) of 26.35, while the positive, negative and overall LRs for preterm preeclampsia were 6.94, 0.34, and 40.07, respectively.

The median PP13 MoMs and 95% CIs varied considerably between the different studies. Comparison of the DELFIA and ELISA studies showed that the DR for all preeclampsia cases at 10% FPR was 78.75% (95% CI: 68.44–88.22) with the ELISA platform and 40.29% (95% CI: 0–61.19) with the DELFIA platform. The positive, negative and overall LRs were 8.25, 0.19 and 53.26 with the ELISA platform and 5.03, 0.55 and 13.29 with the DELFIA platform, respectively. It has also been demonstrated that the ELISA assay of the same samples provides better segregation of PP13 values between preeclampsia cases and controls than the DELFIA assay (166). Among the eight DELFIA assay based studies, good preeclampsia prediction was achieved in two (156, 157), no prediction was achieved in three (155, 159, 162), while varying, moderate prediction was achieved in the rest of the studies. The DELFIA platform differs from the ELISA platform since it includes the capture and detection antibodies in an inverted order, and it utilizes Europium amplification compared to the use of the biotin-extravidin-horse radish peroxidase amplification in the ELISA. These differences may account for some of the differences detected in assay performances (166). However, recent results may suggest that batch differences in the Fab domain of one of the antibodies also play a role in this phenomenon, which is now under examination with the new generation of PP13 kits developed by the “ASPRE” project.

In view of the differential binding of PP13 to cell surfaces containing ABO blood group antigens, and its varying bioavailability in maternal blood depending on the ABO blood type, the adjustment of PP13 MoMs to ABO blood groups further improved their predictive value for preeclampsia as well as for IUGR and the two combined (21). For example, Caucasian and Hispanic women with blood group AB had the lowest, and those with blood group B had the highest first trimester maternal serum PP13 MoMs, while individuals with blood group A or O had intermediate MoMs (21). After adjustment of PP13 MoMs to ABO blood groups, the overall LR for predicting IUGR increased from 2.2 to 5.32, the overall LR for predicting preeclampsia increased from 6.9 to 18.1, and the overall LR for predicting preeclampsia associated with IUGR increased from 5.6 to 27.9.

Earlier studies have shown increased accuracy for the prediction of severe cases of preeclampsia over the mild ones (152, 154, 157). Based on these findings, the large differences in prediction accuracy demonstrated in the meta-analysis can probably be attributed to the differences in the severity of the included cases. This phenomenon as well as the observation on the reduced first trimester PP13 MoMs after IVF (164) are under further examination by the “ASPRE” project, which targets the longitudinal, multi-center examination of 33,000 maternal blood specimens.

### Performance of first trimester PP13 as part of a multiple marker panel

A growing body of evidence suggests that the prediction of preeclampsia can be improved using multi-parametric approaches, combining data derived from multiple biomarkers (153, 165). Initially, PP13 was evaluated as a single marker with MoMs adjusted to various pregnancy features as detailed above. It was then evaluated over a background risk calculated according to preeclampsia in a previous pregnancy, medical history of gestational diabetes, kidney and cardiovascular diseases, maternal age, ethnicity, BMI and conception by assisted reproduction techniques. This analysis showed that the sensitivity of PP13 for predicting all cases of preeclampsia increased from 52 to 59% at 10% FPR after combining with background risk factors (167). Subsequently, PP13 and background risk factors were also combined with the mean arterial pressure (MAP), which further increased the detection rate to 93% for all cases of preeclampsia at 10% FPR (167). Combining PP13 with placental growth factor (PlGF) (156) or with additional biochemical markers [i.e. pregnancy associated plasma protein A (PAPP-A), PlGF and ADAM metallopeptidase domain 12 (ADAM12)] were also accompanied by an increased DR for preeclampsia in spite of the varying predictive values of the individual biomarkers (165). In seven studies, the risk prediction was based on combining PP13 and uterine artery Doppler pulsatility index (PI), which also showed increased prediction accuracy (148, 150, 154, 157, 165, 168, 169). Comprehensive risk algorithms were further developed based on a combined multi-marker analysis that took into consideration the background risk (as detailed above), MAP, Doppler PI, and a panel of serum biomarkers. This approach yielded much higher predictive value and accuracy than individual markers (157), especially for early-onset (<34 weeks) and preterm (<37 weeks) preeclampsia. This is consistent with the results of several other studies that used combined biomarker panels and various types of risk prediction algorithms to obtain better risk prediction (170–172). It was therefore concluded that the introduction of a broad biomarker panel for the evaluation of preeclampsia and other maternal and fetal pregnancy disorders could present a change in deploying antenatal care as formulated by the inverted pyramid model of perinatal evaluation in pregnancy (173). In agreement with these, the combination of PP13, Doppler PI, MAP (or maternal artery stiffness) increased the DR of preeclampsia to 93% for early-onset preeclampsia and to 86% for all preeclampsia cases at 10% FPR (174). This preeclampsia prediction accuracy satisfies the World Health Organization (WHO) requirements for the clinical introduction of a disorder predicting procedure in terms of clinical usefulness in disease management and disorder prevention (175, 176).

### Longitudinal assessment of PP13 in maternal blood

A repeated measure of a marker level was identified as a better method to get a more accurate prediction of the risk to develop pregnancy disorders, initially for Down syndrome (177) and also for preeclampsia (178, 179), or for IUGR and preeclampsia combined (180). A large study on PP13, which utilized repeated measures in the first, second and third trimesters, provided increased prediction accuracy compared to the first trimester test alone, either when a contingent or a combined model was used (151). This was further confirmed in a smaller study using repeated measures of PP13 every 2–4 weeks (37, 147).

The significance of the repeated measure of PP13 is also high since gestational age-related changes in maternal blood PP13 concentrations are very different between normal pregnancy and those with preeclampsia. While in patients who develop preeclampsia PP13 concentrations are lower in the first trimester than in normal pregnant women, the use of repeated measures of PP13 in longitudinal or cross-sectional studies showed that PP13 concentrations sharply increase in preeclampsia patients between the first and third trimesters compared to the moderate change that can be observed in women with normal pregnancy. The most prominent increase is seen when preeclampsia enters into the clinico-pathological stage. For example, between the first and third trimesters, maternal PP13 MoMs were detected to increase by ~1.5 to 3-fold in normal pregnancy compared to the 3.5 to 7.7-fold increase in preeclampsia, and occasionally even more (21, 36, 37, 98, 151, 181). Interestingly, the slope of increase was different among individuals, and it seemed to be related to patient characteristics like obesity, ethnicity, maternal age, parity, and particularly the severity of the disease.

Of importance, when taking into account the effect of ABO blood groups on the longitudinal changes in PP13 across the three trimesters, the regression slope of PP13 concentrations and MoMs were steeper in blood group B than in blood groups A and O, but not in blood group AB. The characteristic changes during gestation in serum PP13 concentrations in women with different ABO blood groups inversely mirrored the relative binding of PP13 to various ABO blood group erythrocytes, suggesting a dynamic change in PP13 sequestration on erythrocyte surfaces depending on gestational age and actual PP13 concentrations (Figure 3C) (21).

As described before, there is an increased shedding of PP13-rich syncytiotrophoblastic microvesicles from placental villi when women enter into the clinico-pathological stage of preterm preeclampsia (36, 37). Importantly, it was proposed that these microvesicles release their PP13 content, leading to the increased maternal blood PP13 concentrations in these cases (36, 37, 73). Since the extent of microvesicle shedding is related to the extent of placental ischemic stress and it is significantly more pronounced in severe cases of preeclampsia, particularly with preterm than with term onset (182), the longitudinal slope of changes in PP13 could be used as an additional parameter to predict case severity (36, 151). This phenomenon explains why so much difference was found in early-onset or preterm preeclampsia in PP13 compared to gestational-age matched controls in the third trimester.

## Meta-Analysis for the Prediction of the Risk for Preeclampsia with Third Trimester PP13

### Methods and included studies

Because of the increased PP13 concentrations in preeclampsia in the third trimester, it was hypothesized that PP13 testing can be further utilized for the prediction and diagnosis of preeclampsia during this period. To address this question, we have conducted a meta-analysis on third trimester datasets and found studies that utilized the PP13 ELISA but not DELFIA platform. The PP13 ELISA utilizes a pair of anti-PP13 mouse monoclonal antibodies (27-2-3 and 215-28-3 MAbs) that were selected based on their high (10^–9^ M) affinity to native and recombinant PP13 (5, 71). As a result, the detection limit of the ELISA was 3–8 pg/ml, the linear detection range was between 12.5–400 pg/ml, and the kit-to-kit, operator-to-operator and batch-to-batch variations were between 3–12% (5).

From the 71 studies published on PP13, the current meta-analysis identified eight clinical studies published between August 2008 and March 2014 that contained third trimester maternal blood PP13 data. These had an international scope involving Israel (71, 98, 151), Hungary (36), Austria (37, 181) and the USA (21). These studies were either performed as part of longitudinal clinical trials or cross-sectional studies that focused on the ELISA-based evaluation of PP13 between 26 and 40 weeks of gestation. In all studies, the ROC curves were based on PP13 adjusted to gestational week specific MoMs, which were further adjusted to BMI, smoking, ethnicity, maternal age as well as parity. In one study, PP13 values were further adjusted to the ABO blood groups (21). In a very recent study the adjustment was further made to conception by IVF (167).

The meta-analysis was performed by a Forest plot method (183). There were three occasions in which the determination of PP13 in maternal blood was extracted from studies performed in separate time points, which were included as separate studies (36, 181, 184). Accordingly, the dataset for the analysis was based on 11 cohorts. The DR at 10% FPR was extracted from the published ROC curves or by communicating with the authors and obtaining complementary data. The 95% CI of the DR was extracted or calculated from ROC curves of the published study or by using the web-calculator^2^. The analysis pooled clinical results from all singleton pregnancy studies, irrespective whether these were prospective cohort studies or case-control studies that enrolled low- or high-risk patients or all-comers. The pooled dataset included all preeclampsia cases and then a sub-analysis was performed for preterm and early-onset preeclampsia cases. Preeclampsia associated with IUGR and/or HELLP syndrome was compared to all preeclampsia cases, but there were too few cases with these additional complications to enable a true meta-analysis.

Regarding the detection level, the meta-analysis has also evaluated the overall LR of developing preeclampsia by dividing the positive LR with the negative LR as described earlier. The overall LR calculation took into consideration the relative weight of each of the cohorts in terms of study size and the number of women with preeclampsia.

### Results of the meta-analysis

In total, 2,750 third trimester pregnant women were tested. 193 women subsequently developed preeclampsia out of whom 30.7% had preterm preeclampsia and 7.6% had early-onset preeclampsia. All but one study enrolled all-comers, and only one study enrolled high-risk patients. In this latter study the correlation between having prior risk of preeclampsia based on major risk factors and a high level of PP13 in the third trimester was low (*R* = 0.13), indicating that the two are independent evaluators (167).

**Figure 10:**
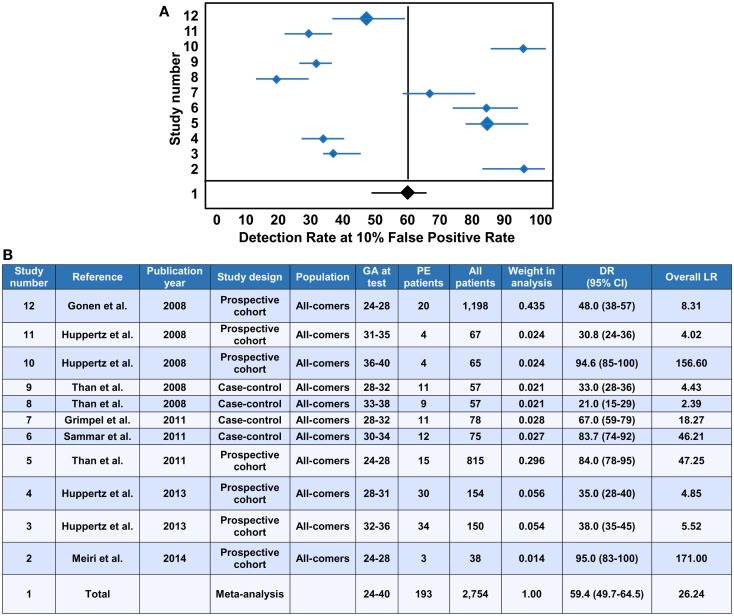
**Meta-analysis of PP13 in predicting preeclampsia in the third trimester**. **(A)** A Forest plot analysis was performed including 11 studies based on unaffected and all preeclampsia cases. The detection rate (DR) at 10% False Positive Rate (FPR) of all cases of preeclampsia is shown in case-control and prospective cohort studies using all-comers. The DR was extracted from Receiver Operation Characteristics (ROC) curves based on the adjusted multiple of the medians (MoMs) of PP13.The final analysis took into consideration the total study size and the size of the preeclampsia group. Number 1 on the study list reflects the results of the meta-analysis depicted with a dark filled diamond compared to individual studies depicted with blue diamonds. The relative weight of a certain study in the analysis is reflected by the relative size of the diamonds. **(B)** The table lists all studies used to perform the Forest plot for the meta-analysis. Weight represents the relative impact of the study in the meta-analysis. DR for 10% FPR is shown along with the 95% confidence interval (95% CI). The Likelihood ratio (LR) was calculated for positive LR [sensitivity/(1-specificity)], negative LR [(1-sensitivity)/specificity] and overall LR (positive LR/negative LR). For the meta-analysis, the values were adjusted to the relative weight of each study in the meta-analysis. The numbers on the left side of the table correspond to the graph numbers in **(A)**. PE, preeclampsia; GA, gestational age in weeks.

**Figure 11:**
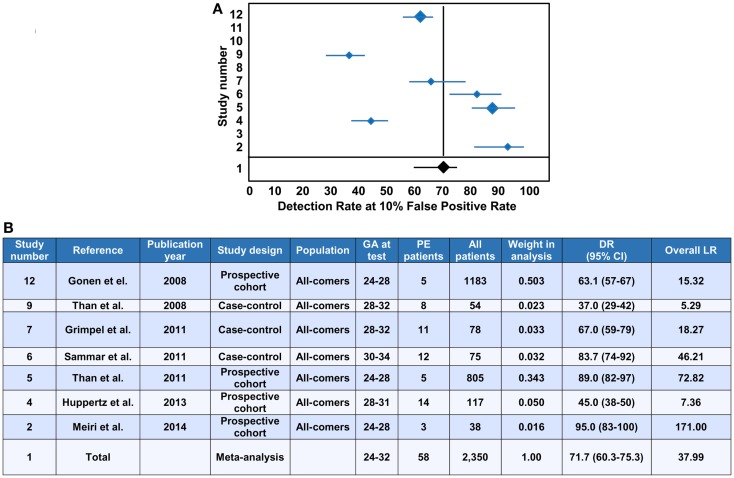
**Meta-analysis of PP13 in predicting preterm preeclampsia in the third trimester**. **(A)** Forest plot analysis was performed including seven studies based on unaffected and preterm preeclampsia cases. The detection rate (DR) at 10% False Positive Rate (FPR) of cases of preterm preeclampsia is shown in case-control and prospective cohort studies using all-comers. The DR was extracted from Receiver Operation Characteristics (ROC) curves based on the adjusted multiple of the medians (MoMs) of PP13. The final analysis took into consideration the total study size and the size of the preeclampsia group. Number 1 on the study list reflects the results of the meta-analysis depicted with a dark filled diamond compared to individual studies depicted with blue diamonds. The relative weight of a certain study in the analysis is reflected by the relative size of the diamonds. **(B)** The table lists studies used to perform the Forest plot for the meta-analysis of cases of preterm preeclampsia <37 weeks), including early onset preeclampsia (<34 weeks). Weight represents the relative impact of the study in the meta-analysis. DR for 10% FPR is shown along with the 95% confidence interval (95% CI). The Likelihood ratio (LR) was calculated for positive LR [sensitivity/(1-specificity)], negative LR [(1-sensitivity)/specificity] and overall LR (positive LR/negative LR). For the meta-analysis, the values were adjusted to the relative weight of each study in the meta-analysis. The numbers on the left side correspond to the graph numbers in **(A)**. PE, preeclampsia; GA, gestational age in weeks.

In all studies, maternal blood PP13 MoMs were higher in women who subsequently developed preeclampsia compared to unaffected women although the variation between individual data-points within a study or between studies was large. Therefore, the sensitivity of using PP13 as a biomarker for predicting the risk of the subsequent development of preeclampsia had a broad range (14–100%). The mean DR at 10% FPR for all preeclampsia cases was 59.4% (95% CI: 49.7–64.5) (Figures 10A,B). The DR of PP13 for preterm preeclampsia (which included all early-onset preeclampsia cases) was 71.7% (95% CI: 60.3–75.3) (Figures 11A,B). Since there were few studies with data from patients with early-onset preeclampsia, this separate analysis had insufficient power for statistical analysis.

The time of detection ranged between 28–32 to 36–40 weeks, and the evaluation of the DR per gestational week yielded a regression line of Y = 1.3986X + 100.58, where X was the gestational week and the regression coefficient (*R*) was 0.2. These results have indicated that the variations are indeed independent of the gestational week at testing. When evaluated according to the correlation with MAP or urine protein, the DR appeared to be related to the severity of the cases in a given study, with regression coefficient values of 0.61 and 0.73, respectively. This means that the higher the hypertension and proteinuria, the higher the third trimester PP13 MoMs in maternal blood, and the better the prediction. A combined algorithm of PP13, MAP and proteinuria, which was available for nine out of the 11 studies, yielded a 95% DR for preterm preeclampsia and 85% for all preeclampsia at 5% FPR, showing the value of combining all parameters (data not shown). In conclusion, the meta-analysis indicates that higher third trimester maternal blood PP13 among women who will subsequently develop preeclampsia reaches the clinical diagnostic level.

The positive LR for all cases of preeclampsia in the meta-analysis was 5.94 and the negative LR was 0.45, providing an overall LR of 26.24 (Figure 10B). The positive LR for preterm preeclampsia in the meta-analysis was 7.17 and the negative LR was 0.31, providing an overall LR of 37.99 (Figure 11B). These LRs are lower compared to the overall LRs of first trimester PP13, but these can still be considered respected LRs by the criteria of the WHO (176).

Reduced blood concentrations of PIGF in the third trimester have been suggested for predicting the symptoms of preeclampsia within 14 days of the test. This fast and quantitative TRIAGE test, measuring the decreased PlGF concentrations in maternal blood, also predicts the anticipated disease severity (185, 186). Of interest, the combination of anti-angiogenic factors and PlGF (or their ratio) increase the prediction rate of severe late-onset preeclampsia in the third trimester (179). Combining low PIGF with high PP13 maternal blood concentrations may generate an even better test. Thus, it is essential to investigate the PP13/PIGF ratio as a better diagnostic tool for preeclampsia in the third trimester. This will be further explored by the “ASPRE” project, in which at least 1,500 high-risk patients out of the 33,000 enrolled pregnant women will be tested longitudinally in the first, second and third trimesters of pregnancy.

## Summary and Conclusions

**Figure 12:**
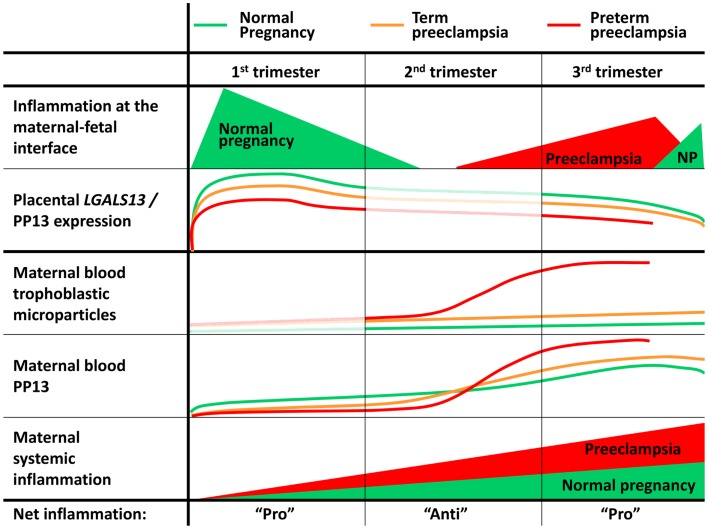
**PP13 expression is related to inflammatory changes at the maternal-fetal interface and in maternal circulation**. This summary figure consolidates the results of numerous studies related to various facets of PP13 in normal pregnancies and in preeclampsia. The placental expression of *LGALS13* and PP13 is strong in the first trimester, and the secreted protein can be detected in maternal blood from gestational weeks 5 to 6 in normal pregnancies. From the decidual veins PP13 gets into the decidua, where it is deposited extracellularly or phagocytosed, coincident with maternal immune cell infiltration and the formation of the “Zones of Necrosis” (ZONEs) adjacent to the decidual veins. Although maternal serum PP13 concentrations do not change, the relative placental expression and decidual deposition of PP13 declines until the end of the first trimester in parallel with the decrease in the number of ZONEs. In the second and third trimesters, maternal serum PP13 concentrations rise due to the growing number of villi and trophoblast volume in the placenta, paralleling the escalation in maternal systemic inflammation. In preeclampsia, especially in early-onset cases, there are lower placental expression and maternal serum concentrations of PP13 in the first trimester, coincident with impaired trophoblast invasion and spiral artery remodelling. Starting from the second trimester, ischemic placental stress and pro-inflammatory changes at the maternal-fetal interface are also reflected by the increased shedding of aponecrotic microvesicles, which carry a considerable amount of PP13, elevating maternal blood PP13 concentrations. PP13 expression in the first trimester is associated with inflammation at the maternal-fetal interface. Similarly, maternal blood PP13 concentrations in the second and third trimesters parallel maternal systemic inflammation. As a consequence, PP13 has a good diagnostic value for the prediction and diagnosis of preeclampsia in the first and third trimesters. NP: normal pregnancy.

Galectins are glycan-binding proteins that regulate innate and adaptive immune responses, and some galectins confer maternal-fetal immune tolerance in eutherian mammals. A chromosome 19 cluster of galectin genes has emerged in anthropoid primates, species with deep placentation and long gestation, in which this galectin network may confer additional immunoregulatory functions to enable deep placentation. These cluster galectins, including PP13, have a conserved structure, CRD and sugar-binding preference resembling other mammalian galectins. PP13 is solely expressed by the human placenta, predominantly by the syncytiotrophoblast, from where it is released into the maternal blood. PP13 expression and release from the individual placental villi is highest in the first trimester when maternal immune cell infiltration into the decidua is at its peak (Figure 12) to promote successful placentation including embryo implantation, trophoblast invasion, repair of the uterine epithelium and removal of cellular debris. Of interest, PP13 released from the villi is deposited around uterine veins and contributes to the formation of “ZONEs” of apoptotic and necrotic immune cells, which peak parallel with the start of spiral artery remodelling in the first trimester. Because PP13 is capable of inducing the apoptosis of activated T cells and the cytokine production of macrophages, it was postulated that these PP13 deposits in the decidual extracellular matrix may attract maternal immune cells away from the sites of maternal spiral artery formation to the decidual veins, and may promote a tolerogenic environment that facilitates trophoblast invasion and placentation. How important the roles of PP13 are during early placentation may be well reflected by observations showing decreased placental expression and maternal serum concentrations of PP13 in the first trimester in preeclampsia (Figure 12), a syndrome originating from severely impaired trophoblast invasion and placentation. Moreover, mutations in the promoter and in the exons of *LGALS13* presumably leading to altered, misfolded or non-functional protein expression are associated with a higher frequency of preeclampsia and also other obstetrical syndromes which involve immune dysregulation.

PP13 maternal blood concentrations steeply increase in preeclampsia compared to normal pregnancy starting in the second trimester, with the steepness correlated to disease severity. This phenomenon is closely related to the ischemic placental stress and the consequent increase in trophoblastic shedding of PP13 immunopositive microvesicles (Figure 12). Because of the pro-inflammatory nature of these aponecrotic trophoblast microvesicles and other “toxins” released from the placenta, preeclampsia, especially its early-onset subform, is characterized by an exaggerated maternal systemic inflammation and generalized endothelial dysfunction, leading to kidney damage, proteinuria and hypertension. It is interesting that reduced placental PP13 expression in preeclampsia correlates with altered immune-interactions at the maternal-fetal interface. Similarly, maternal blood PP13 concentrations in the second and third trimesters are elevated in relation to the increased placental stress and maternal systemic inflammation (Figure 12). These phenomena have already been utilized for developing a PP13 blood test for predicting preeclampsia, and indirectly for impaired placentation, in the first trimester. The analysis provided here shows that this test may be further used for preeclampsia diagnosis in the third trimester.

Functional studies have just started to assess the *in vitro* and *in vivo* effects of PP13 during pregnancy, showing various functions that PP13 may have at the maternal-fetal interface. *In vitro* studies need to take into account the pleiotropic actions of PP13, which may depend on the activation and differentiation status of the affected cells, the way PP13 is released from the placenta (e.g. free or extracellular vesicle-bound), the redox status of the environment, and the interaction of PP13 with various small molecules. *In vivo* studies, while starting in rodents, may eventually need to be extended to other models, optimally to primates. Nevertheless, the results of the first studies support the importance of PP13 in the regulation of blood pressure and vascular remodelling at the maternal-fetal interface.

## Conflict of Interest Statement

Hamutal Meiri and Sveinbjorn Gizurarson hold a patent for the use of the PP13 supplement to treat preeclampsia. All other co-authors have no conflict of interest.

## Footnotes

^1^www.1000genomes.org

^2^http://www.causascientia.org/math_stat/ProportionCI.html
